# Supplementary material for: COVID-19 Vaccine Effectiveness and Risk Factors of Booster Failure in 480,000 Patients with Diabetes Mellitus: A Population-Based Cohort Study
Source: Microorganisms. 2025 Apr 24;13(5):979. doi: 10.3390/microorganisms13050979 (PMC12114578; doi:10.3390/microorganisms13050979)
Supplement: Supplementary file 1 [file microorganisms-13-00979-s001.zip › microorganisms-3536804-supplementary.pdf]

Supplementary Material – Microorganisms

COVID-19 vaccine effectiveness and risk factors of booster failure in 480,000 patients with diabetes mellitus: A population-based cohort study

Authors

Maria Christina L. Oliveira, Daniella R. Martelli, Ana Cristina Simões e Silva, Cristiane S. Dias, Lilian M. Diniz, Enrico A. Colosimo, Clara C. Pinhati, Stella C. Galante, Fernanda N. Duelis, Laura E. Carvalho, Laura G. Coelho, Maria Eduarda T. Bernardes, Hercílio Martelli-Júnior, Fabrício E. Oliveira, Robert H. Mak, and Eduardo A. Oliveira

-

Contents

Supplementary Material (S1)

Supplementary Material (S2)

Supplementary Table S1

Supplementary Material (S1)

SIVEP-gripe (The Influenza Epidemiological Surveillance Information System)

The participants in this study were patients who had been hospitalized with severe acute respiratory infection (SARI) and were recorded in the Influenza Epidemiological Surveillance Information System (SIVEP-Gripe) database, an official database maintained by the Ministry of Health for monitoring severe acute respiratory syndrome (SARS) cases in Brazil. The Influenza Epidemiological Surveillance Information System, SIVEP-Gripe (*Sistema de Informação de Vigilância Epidemiológica da Gripe*), was established by the Brazilian Ministry of Health (MS) in 2009, due to the Influenza A (H1N1) pandemic.

SARI is a notifiable disease in Brazil, and it must be reported to health authorities within 24 hours of suspicion. Therefore, all Brazilians individuals treated in a public or private health system suspected of SARI must be registered in the databases provided by the Ministry of Health. For hospitalized cases, data are registered in the SIVEP-gripe database which is updated weekly. Consequently, notification is compulsory, and this information is recorded in the SIVEP-Gripe database, which receives data from public and private hospitals, emergency care units (UPAs), mobile emergency medical services (SAMU), death verification services (SVO), and Municipal Health Departments. Upon admission, patients were tested for SARS-CoV-2 and other common seasonal respiratory viral etiologies using reverse transcriptase-polymerase chain reaction or antigen testing.

Database Management

The Brazilian Ministry of Health makes available these databases in the platform OpenSUS (https://opendatasus.saude.gov.br/dataset). Therefore, detailed information regarding these databases, including reporting form and data dictionary, codes, and all de-identified data, such as individual participant data, are publicly available at this website. For the present study, we downloaded the last version available of the datasets in April 2023. For the purpose of the present analysis, we limited the period of the study from February 2020 to February 2023.

Supplementary Material (S2)

Vaccination program

In Brazil, the Ministry of Health (MS) is the only provider of COVID-19 vaccines. Following the Brazilian National Health Surveillance Agency (ANVISA) issued emergency use authorization, four vaccine schedules of different platforms were authorized in Brazil for the adult population: mRNA vaccine (Pfizer–BioNTech), virus-inactivated vaccine (Sinovac; CoronaVac); and viral-vector vaccines (ChAdOx1 nCoV-19, Astrazeneca and Ad26.COV2.S, Johnson & Johnson's Janssen).

The COVID-19 vaccination program in Brazil was sequentially implemented according to the following chronology:

12/16/2020 - National Operational Plan against Covid-19 (PNO) - First edition-

The plan defined the priority groups: health workers (including health professionals, support professionals, caregivers for the elderly, among others), institutionalized people aged 60 and over, elderly population (60 and over), indigenous people living in demarcated lands villages, traditional isolated communities, homeless population, people with morbidities (Diabetes mellitus;, severe arterial hypertension; chronic obstructive pulmonary disease; kidney disease; cardiovascular and cerebrovascular diseases; transplanted individuals solid organ disease; sickle cell anemia; grade III obesity), education workers, people with severe permanent disabilities, members of the security and rescue forces, employees of the deprivation of liberty system, public transport workers, road freight transporters, population deprived of liberty.

01/18/2021 – Start of vaccination campaign against COVID-19 in Brazil following the recommendations of rollout plan.

05/27/2021 - Beginning of vaccination of the general population (18 to 59 years of age) and the continuity of vaccination of priority groups listed in the National Plan of Operationalization of Vaccination against Covid-19 (PNO).

02/09/2021 - Included children and adolescents between 12 and 17 years old, as well as pregnant women, postpartum women and breastfeeding women without comorbidities in the target audience for vaccination against COVID-19.

09/20/2021 - the Technical Chamber on Covid19 Immunization (CTAI COVID-19), included a a booster dose of the vaccine for all elderly people over 70 years of age, which should be administered 6 months after the last dose of the vaccination schedule (second dose or single dose), regardless of the vaccine administered. The booster dose was also extended for people with a high degree of immunosuppression.

11/17/2021 – Extended administration of a booster dose of Covid-19 vaccines to people over 18 years of age.

03/23/2022 - Recommendation of the second booster dose of vaccines against covid-19 in people aged 80 or over.

05/02/2022 - Recommendation of the second booster dose of vaccines against covid-19 in people aged 70 or over and in institutionalized people aged 60 or over .

05/19/2022 - Recommendation of the second booster dose of vaccines against covid-19 in people aged 60 or over.

03/06/2022 - Recommendation of the second booster dose of vaccines against covid-19 in people aged 50 or over.

06/03/2022 - Recommendation of the second booster dose of vaccines against covid-19 for healthcare workers.

06/20/2022 - Recommendation of the second booster of covid-19 vaccines in people aged 40 years and over.

12/27/2022 – Recommendation that the vaccination of people under 40 against covid-19 occurs preferably with vaccines from platforms that are not viral vector.

04/24/2023 - Expansion of the recommendation for the bivalent covid-19 vaccine as a booster dose for all people aged 18 years or over.

To give reliable information regarding the vaccination program in Brazil, the Ministry of Health updated the SIVEP-Gripe database in 2021, including the “vacina_covid” field, with the respective dates for the first dose and subsequent ones. From this field, we created for our analysis closed fields related to the vaccination status (boosted [3 doses], fully vaccinated [2 doses], partially vaccinated [1 dose], unvaccinated).

Supplementary Table S1. Univariate analysis of risk factors of death among patients hospitalized with laboratory-proven SARS-CoV-2 symptomatic infection

Covariates[a]	Death (%) 681,681	Survival (%) 1,449,408	Unadjusted OR	95% Confidence interval	P
Cohort					
Without comorbidities	705,799 (22.5)	205,471 (77.5)	Reference		
Diabetes	67,905 (32,7)	32,944 (67.3)	1.66	1.64 – 1.69	<0.001
With comorbidities	459,048 (37,7)	278,094 (62.3)	2.08	2.06 – 20.9	<0.001
Diabetes with comorbidities	216,656 (43,3)	165,172 (56.7)	2.62	2.59 – 2.64	<0.001
Age group (years)					
18 - 29	9,678 (10,3)	84,494 (89,7)	Reference		
30 - 59	196,303 (20,4)	767,954 (79,6)	2.23	2.18 – 2.28	<0.001
60 – 79	314,747 (40,7)	457,718 (59,3)	6.00	5.87 – 6.13	<0.001
> 80	160,953 (53,6)	139,242 (46,4)	10.09	9.87 – 10.3	<0.001
Sex (n = 2,131,073)					
Female	301,072 (31,4)	657,234 (68,6)	Reference		
Male	380,603 (32,5)	792,164 (67,5)	1.049	1.04 – 1.05	<0.001
Region					
Southeast	334,270 (31,8)	717,950 (68,2)	Reference		
South	106,967 (29,5)	256,197 (70,5)	0.89	0,89 - 0,86	<0.001
Central-West	61,287 (28,4)	154,572 (71,6)	0.85	1,18 - 1,19	<0.001
Northeast	128,036 (35,6)	231,222 (64,4)	1.19	1,21 - 1,24	<0.001
North	51,121 (36,4)	89,467 (63,6)	1.23	1.15 - 1.29	<0.001
Ethnicity (n = 1,739,886)					
White	294,601 (32,4)	613,770 (67,6)	Reference		
Brown	240,781 (33,6)	474,806 (66,4)	1.06	1.05 - 1.06	<0.001
Black	34,625 (38,1)	56,314 (61,9)	1.28	1.26 - 1.29	<0.001
Asian	6,490 (30,7)	14,684 (69,3)	0.92	0.89 - 0.95	<0.001
Indigenous	1,396 (36,6)	2,419 (63,4)	1.20	1.13 - 1.28	<0.001
Educational level (n = 754,185)					
Illiterate	25,734 (51,3)	24,385 (48,7)	Reference		
Elementary	88,211 (43,1)	116,397 (56,9)	0.72	0.70 - 0.73	<0.001
Middle-School	51,317 (36,5)	89,119 (63,5)	0.54	0.53 - 0.56	<0.001
High-School	67,276 (27,5)	177,804 (72,5)	0.36	0.35 - 0.37	<0.001
College	27,486 (24,1)	86,456 (75,9)	0.30	0.29 - 0.31	<0.001
Oxygen saturation <95% (n = 1,763,683)					
No	94,830 (21,7)	94,830 (21,7)	Reference		
Yes	477,408 (36,0)	477,408 (36,0)	2.03	2.02 – 2.05	<0.001
Number of comorbidities (n = 1,648,412) *					
None	205,471 (22,5)	705,799 (77,5)	Reference		
1	181,163 (34,4)	345,659 (65,6)	1.80	1.79 - 1.81	<0.001
2	75,486 (44,2)	95,430 (55,8)	2.72	2.68 - 2.75	<0.001
≥3	21,445 (54,4)	17,959 (45,6)	4.10	4.02 - 4.18	<0.001
SARS-CoV-2 strain					
Ancestral	228,688 (32,8)	467,575 (67,2)	Reference		
Gamma	347,279 (32,2)	732,504 (67,8)	0.97	0.96 - 0.98	<0.001
Delta	34,367 (29,9)	80,668 (70,1)	0.87	0.86 - 0.88	<0.001
Omicron	71,347 (29,7)	168,661 (70,3)	0.86	0.86 - 0.87	<0.001
Vaccine doses (n = 1,887,890)					
None	1,000,061(67,6)	479,013 (32,4)	Reference		
One	66,784 (64,4)	36,990 (35,6)	1.15	1.14 - 1.17	<0.001
Two	134,154 (67,2)	65,372 (32,8)	1.02	1.00 - 1.03	<0.001
Three	81,727 (77,5)	23,789 (22,5)	0.61	0.59 - 0.62	<0.001
Nosocomial infection					
No	664,029 (31,8)	1,425,679 (68,2)	Reference		
Yes	17,652 (42,7)	23,729 (57,3)	1.60	1.57 – 1.63	<0.001
Admission Year					
2020	228,688 (32,8)	467,575 (67,2)	Reference		
2021	385,786 (31,9)	822,096 (68,1)	0.96	0.95 - 0.97	<0.001
2022	65,884 (29,9)	154,488 (70,1)	0.87	0.86 - 0.88	<0.001
2023	1,323 (20,1)	5,249 (79,9)	0.51	0.48 - 0.55	<0.001

[a]Data (n) in the first column represents the available sample for covariates with missing values

* Excluded patients with diabetes
